# Morphological Responses and Gene Expression of Grain Amaranth (*Amaranthus* spp.) Growing under Cd

**DOI:** 10.3390/plants9050572

**Published:** 2020-04-30

**Authors:** Veronika Lancíková, Marián Tomka, Jana Žiarovská, Ján Gažo, Andrea Hricová

**Affiliations:** 1 Institute of Plant Genetics and Biotechnology, Plant Science and Biodiversity Centre, Slovak Academy of Sciences, Nitra 95007, Slovakia; veronika.lancikova@savba.sk; 2 Department of Biochemistry, Faculty of Biotechnology and Food Sciences, Slovak University of Agriculture, Nitra 94976, Slovakia; marian.tomka@uniag.sk; 3 Department of Genetics and Plant Breeding, Faculty of Agrobiology and Food Resources, Slovak University of Agriculture, Nitra 94976, Slovakia; jana.ziarovska@uniag.sk (J.Ž.); jan.gazo@uniag.sk (J.G.)

**Keywords:** cadmium stress, grain amaranth, stress-related genes, phytoremediation potential

## Abstract

Phytoremediation efficiency depends on the ability of plants to accumulate, translocate and resist high levels of metals without symptoms of toxicity. This study was conducted to evaluate the potential of grain amaranth for remediation of soils contaminated with Cd. Three grain amaranth varieties, “Pribina” (*A. cruentus*), “Zobor” (*A. hypochondriacus x A. hybridus*) and Plainsman (*A. hypochondriacus x A. hybridus*) were tested under different level of Cd (0, 5, 10 and 15 mg/L) in a hydroponic experimental treatment. All could be classified as Cd excluders or Cd-hypertolerant varieties able to grow and accumulate significant amounts of Cd from the hydroponic solution, preferentially in the roots. Under the highest level of Cd exposure, qRT-PCR expression analysis of five stress-related genes was examined in above- and below-ground biomass. The results show that the Cd concentration significantly increased the mRNA level of chitinase 5 (*Chit 5*) in amaranth roots as the primary site of metal stress. The involvement of phytochelatin synthase (*PCS1*) in Cd detoxification is suggested. Based on our findings, we can conclude that variety “Pribina” is the most Cd-tolerant among three tested and can be expected to be used in the phytomanagement of Cd loaded soils as an effective phytostabiliser.

## 1. Introduction 

Soil contamination by heavy metals (HMs) has become a serious threat to the human population, agriculture and food security. Anthropogenic activities are significant sources of metals and metalloids in the environment, for instance through rapid industrialisation and urbanisation, mining and agricultural practices such as the uncontrolled application of fertilisers and pesticides [1,2]. The most dangerous are nonessential metals such as cadmium (Cd), lead (Pb), mercury (Hg) and arsenic (As). These elements have no biochemical or physiological function and cause significant damage to living systems, unlike essential metals (Mn, Zn, Fe and Cu) which are beneficial in low concentrations [3]. According to the World Health Organisation, the limit for soil contamination with Cd is 3 mg·kg^−1^ of soil. Worldwide, there are extensive land areas with considerable Cd contamination exceeding the allowed limit, i.e., regions of India [4], Mexico [5], southern Italy [6] and Switzerland [7]. Within the European Union, Tóth et al. [8] investigated the presence of HMs in topsoil samples collected from more than 20,000 locations. This unique soil heavy metal assessment showed that agricultural land of about 137,000 km^2^ (6.24%) would need additional measures and possible remediation.

Remediation methods for heavy metal contaminated areas are based on physical (soil replacement, isolation and vitrification), chemical (soil washing and immobilisation of metals) and biological (phytoextraction, phytostabilisation and phytoevaporation) approaches [9]. Nonbiological methods are effective, but they are also expensive and often damage surrounding areas. In contrast to nonbiological approaches, biological remediation options, employing plants to clean contaminated soils, appear to be environmentally friendly, low-cost and meet sustainability criteria [10,11].

Plants can cope with HM soil contamination in different ways based on their ability to detoxify toxic metals. In general, three groups of plants have been described as follows: a) indicators characterised by their sensitivity to the presence of HMs in soil, b) excluders able to tolerate high loads of HMs without the accumulation of metals and c) hyperaccumulators able to not just tolerate but also accumulate high concentrations of specific HMs [12,13]. The definition of the ideal hyperaccumulator is a fast-growing, high biomass-producing plant with extensive root architecture that accumulates the majority of HMs in shoots [14]. Nowadays, about 500 metal hyperaccumulators have been found within the angiosperms [15,16,17]. Although hyperaccumulation appears to be a preferred characteristic in terms of soil remediation, hypertolerance may represent an interesting solution for the immobilisation of HMs at contaminated sites [18]. The main advantages of phytostabilisation are the establishment of vegetation cover on the surface of contaminated soil, the reduction of HM mobility into water or dust and the stabilisation of soil characteristics such as reduced leaching and soil erosion [18]. It is important to note that hypertolerance and hyperaccumulation are independent mechanisms at the molecular level [13,19].

Several studies have investigated growth responses, metal transportability and accumulation and phytoremoval capability in *Amaranthus* spp. including *Amaranthus hybridus*, *Amaranthus tricolor*, *Amaranthus retroflexus*, *Amaranthus spinosus*, *Amaranthus dubius*, *Amaranthus panuiculatus* were reported earlier [20,21,22,23,24,25]. Research into amaranth responses to Cd have been performed on *Amaranthus mangostanus*, which was used to identify high-Cd and low-Cd cultivars among 23 amaranth cultivars from different ecological regions of China [26]. *Amaranthus hybridus* has been investigated for Cd tolerance, uptake and accumulation [27]; *Amaranthus spinosus* has been assessed for phytoremediation potential [23]. Cadmium phytoextraction by *Amaranthus hypochondriacus* has been reported by Yuan et al. [28] and Guo et al. [29]. Recently, Wang et al. [30] showed an effective increase in Cd uptake and an enhanced ability of *Amaranthus hypochondriacus* plants to absorb and transport Cd from soils using degradable chelators. He et al. [31] suggested a cultivar-dependent capacity for Cd tolerance and accumulation in *Amaranthus gangeticus*.

In the present study, we examined Cd absorption capability as another potential of new grain varieties “Pribina” (*A. cruentus* L.) and “Zobor” (*A. hypochondriacus x A. hybridus*) that are preferentially used for consumption and could be potentially applied in phytoremediation. Their phytoextraction potential was compared to the commercially preferred variety Plainsman (*A. hypochondriacus x A. hybridus*). The main factors related to amaranth performance under Cd stress were examined: (a) the morphological response of amaranth plants to three different Cd concentrations, (b) Cd accumulation capacity into root and shoot tissues determined by Inductively Coupled Plasma Optical Emission Spectometry (ICP-OES) and (c) expression analysis of selected stress-related genes. The results from this study will add to our knowledge about the morphological responses and accumulation potential of grain amaranth to various levels of Cd, and Cd-responsive expression of the genes known to be involved with response processes for (a)biotic stress.

## 2. Results

### 2.1. Amaranth Growth under the Cadmium Exposure

In the present study, the morphological response and phytoextraction capacity of grain amaranth plants “Pribina” (*A. cruentus*), “Zobor” (*A. hypochondriacus x A. hybridus*) and Plainsman (*A. hypochondriacus x A. hybridus*) were investigated in the presence of 0, 5, 10 and 15 mg/L Cd.

The plant response to Cd treatment was manifested by reduced overall plant growth. However, Cd concentrations up to 15 mg/L did not cause any lethal effects. Overall, plant growth was Cd dose-dependent, hence plant fitness at 5 mg/L of Cd was comparable with the control group, but growth was gradually retarded at 10 and 15 mg/L of Cd.

For the “Pribina” and Plainsman varieties, plant dry weight (DW) was reduced by 37.4% and 50.9% in 15 mg/L Cd group compared to the control group, respectively. The root/shoot ratio showed a proportional reduction in root and shoot biomass. In comparison, no reduction in growth was observed for the “Zobor” variety. The same trend was also observed for root and shoot biomass when analysed separately. Plant growth measurements showing fresh/dry weight of control and Cd-treated plants are shown in Supplementary Table S1.

### 2.2. Cadmium Accumulation in Root and Shoot Tissues Determined by ICP-OES

**Figure 1:**
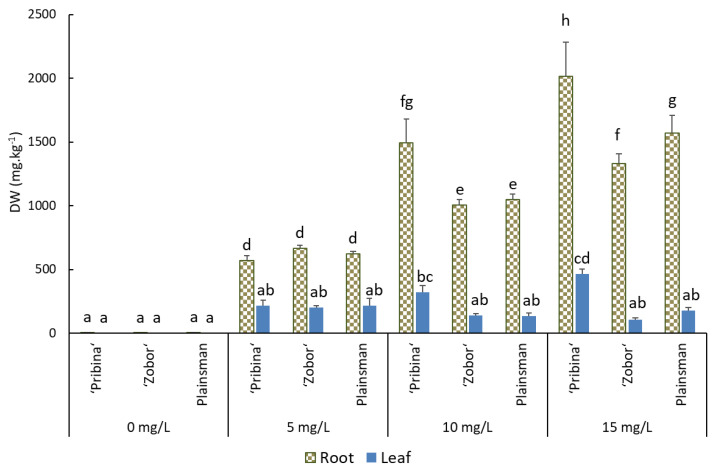
Cadmium uptake into root and shoot tissues (mg/kg^−1^ of DW) of amaranth varieties “Pribina”, “Zobor”, and Plainsman. Cd concentration in both plant tissues was quantified by inductively coupled plasma optical emission spectrophotometer (ICP-OES). Results are the means (±SE; standard error) from five independent biological replicates per each variety and Cd treatment (0, 5, 10 and 15 mg/L). Different letters indicate significant differences between Cd uptake in plant tissues of tested amaranths (LSD test; *p* ≤ 0.05).

ICP-OES analyses revealed that the majority of Cd accumulated in root tissue (Figure 1 and Supplementary Table S1). “Pribina” appeared to have the markedly highest accumulation capacity among the analysed amaranths. The Cd concentration in roots was estimated as 2016.4 ± 593.4 mg·kg^−1^ of DW for the 15 mg/L Cd treatment. In comparison, the Cd concentration in the roots of “Zobor” and Plainsman was determined as 1331.9 ± 172.4 mg·kg^−1^ of DW and 1571.2 ± 306.3 mg·kg^−1^ of DW, respectively. Moreover, significant differences were observed in Cd accumulation in the roots of “Pribina” after 10 and 15 mg/L treatment (*p* ≤ 0.05).

As shown in Figure 1, the Cd absorption in shoots was comparable among varieties, except that the value of “Pribina” at highest Cd treatment was significantly higher (*p* ≤ 0.05). Plant fresh weight after 15 mg/L µM Cd exposure in hydroponic solution was determined as 5.03 ± 1.5, 3.28 ± 0.6 and 1.59 ± 0.21 g for “Pribina”, “Zobor”, and Plainsman, respectively. Considering the fact that nontreated “Pribina” showed higher biomass production compared to “Zobor” and Plainsman, it might be assumed that the Cd accumulation capacity of all the investigated varieties was comparable.

Based on the obtained results, all three varieties may be classified as Cd excluders or Cd-hypertolerant varieties that were able to grow and accumulate significant amounts of Cd from the hydroponic solution.

### 2.3. Translocation of Cadmium from Roots to Shoots 

**Figure 2:**
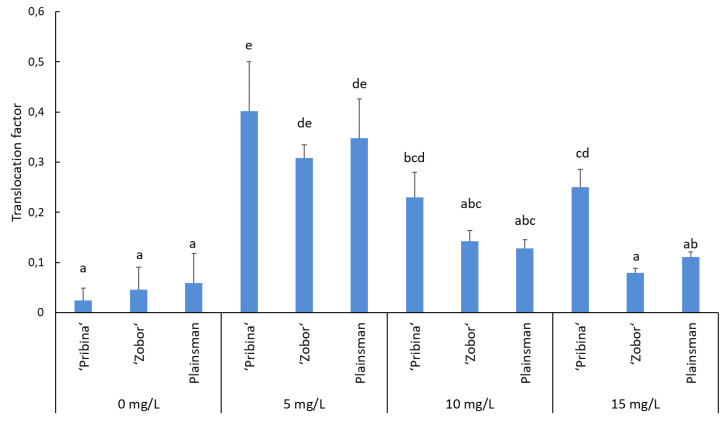
Translocation factor (TF = the relative translocation of metal from roots to shoots expressed in arbitrary units) of cadmium in amaranth varieties “Pribina”, “Zobor”, and Plainsman as influenced by the Cd concentration. Results are the means (±SE; standard error) from five independent biological replicates. Different letters indicate significant differences between gene expression in tested plant tissues (LSD test; *p* ≤ 0.05).

The translocation factor for Cd was significantly higher in “Pribina” than in the hybrid varieties “Zobor” and Plainsman (*p* ≤ 0.05). In “Pribina”, translocation factor (TF) ranged from 0.23 to 0.40 and was the lowest in the medium Cd level (Figure 2). In the case of variety “Zobor”, TF was the highest at the lowest Cd concentration and amounted to 0.31 in comparison with TF = 0.14 and 0.08 obtained at higher Cd concentrations. Translocation of Cd from roots to shoots was comparable in the hybrid commercial variety Plainsman compared to “Zobor”, with TF values decreasing with increased Cd levels (Figure 2). However, at the highest Cd concentration, TF in Plainsman was significantly higher compared to “Zobor” (*p* ≤ 0.05; Figure 2).

A negative correlation was observed between the Cd concentration and TF values, since increasing Cd concentrations resulted in lower TF. The lowest Pearson’s correlation coefficient was estimated for “Pribina” (−0.399; *p* = 0.139), followed by “Zobor” (−0.892; *p* = 0.000) and Plainsman (−0.678; *p* = 0.005).

These results confirmed the excellent ability of “Pribina” to transport Cd from roots to shoots at higher Cd concentrations. However, none of the three analysed amaranths showed a TF greater than one, which would indicate grain amaranth Cd phytoextraction.

### 2.4. Expression Analysis of Stress-Related Genes

**Figure 3:**
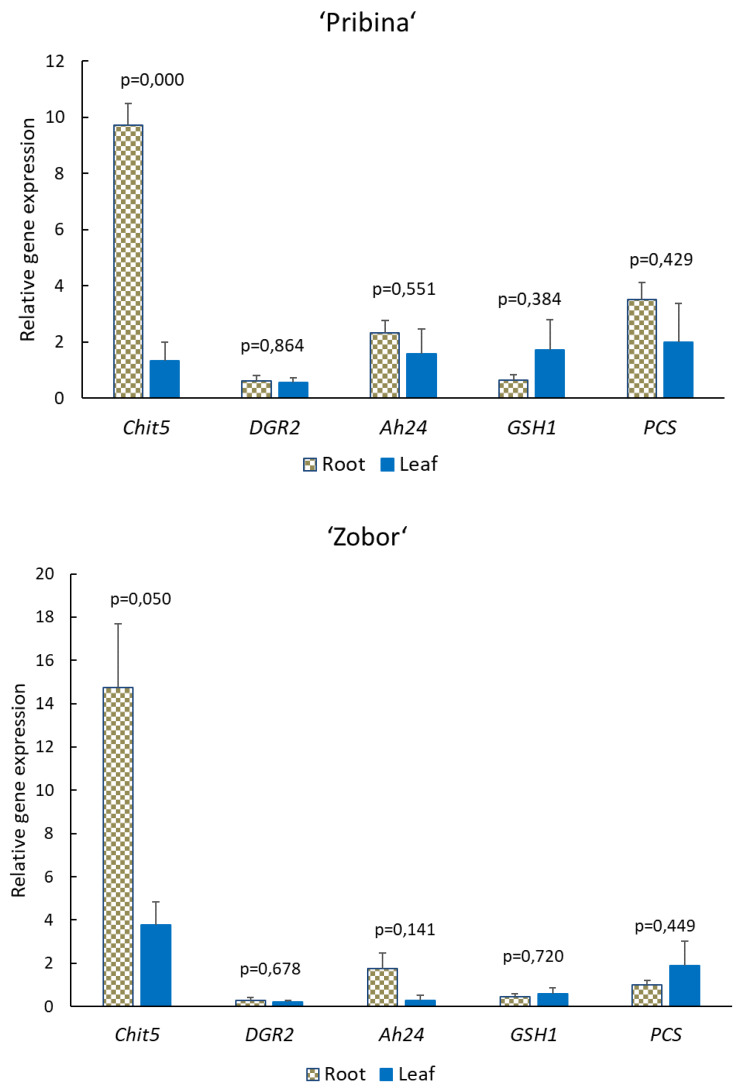

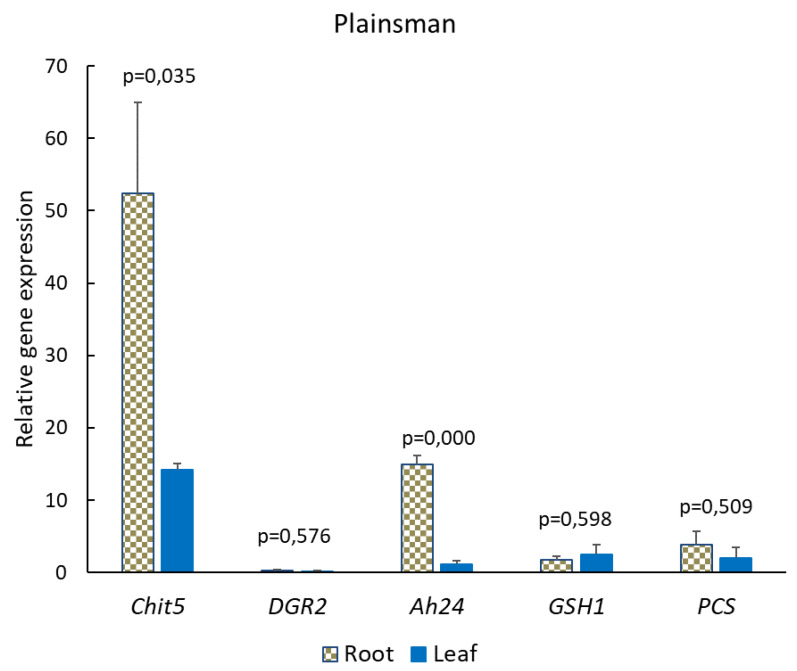
Effect of 15 mg/L Cd treatment on the expression of selected stress-related genes. Quantitative real-time PCR (qRT-PCR) analysis of *Chit5*, *Ah24*, *AhDGR*2, *GSH1* and *PCS* genes were examined in root and leaves of the amaranth varieties “Pribina”, “Zobor” and Plainsman. The expression levels of target genes were quantified with reference to the expression of *A. hypochondriacus actin* (*AhACT*). The relative expression levels are shown as fold changes relative to the copy number of a particular mRNA gene in the control sample. Results are the means (±SE; standard error) from five independent biological replicates. *p*-value represents results of statistical testing between gene expression in roots and leaves by paired t-test.

The expression analysis of the genes *Chit*5, *AhDGR*2, *Ah24*, *GSH1* and *PCS* was performed in both root and leaf tissues after 15 mg/L Cd treatment (Figure 3).

*Chit*5 expression was significantly induced in the roots of all investigated amaranth varieties compared to expression in the leaf tissue (*p* ≤ 0.05). Approximately 9.7, 14.7 and 52.4-fold upregulation was observed in roots of “Pribina”, “Zobor” and Plainsman, respectively. Treatment with 15 mg/L Cd enhanced the expression of *Chit*5 in roots compared to leaves by 7.3, 3.9 and 3.7-fold for “Pribina”, “Zobor” and Plainsman, respectively.

Notably, the gene *Ah24* was most upregulated (15-fold) in the roots of the Plainsman variety with significant differences compared to expression in the leaves (*p* ≤ 0.01). As shown in Figure 3, the transcript level of *GSH1* did not undergo statistically notable alterations between roots and leaves under Cd exposure (*p* > 0.05). The *PCS* expression level was 3.5-fold and 4-fold upregulated in “Pribina” and Plainsman roots, respectively. However, higher expression was observed in the leaves of the Cd-treated “Zobor” variety but without statistical significance (*p* = 0.45).

Interestingly, the *AhDGR*2 gene was downregulated in response to Cd treatment in amaranth tissues. An approximately 40% decrease was noted for “Zobor” and Plainsman roots, while in “Pribina” a 70% decrease was detected. No significant differences between expression in root and leaf tissues were observed (*p* > 0.05). Changes in the expression of the investigated stress-related genes are shown in Figure 3.

## 3. Discussion

Plant species with a large biomass, fast growth, and high accumulation of heavy metals in their tissues without any severe effects are most acceptable for the extensively applicable technique of phytoremediation. Amaranth, as a multipurpose crop, is usually not the first choice plant for the phytoremediation of metal-loaded soils. However, this plant has several important attributes such as a large aboveground biomass, abundant root branches, low demands on growing conditions, and high tolerance against various stress factors.

Inhibited and/or slow growth and low biomass production are the major accompanying characteristics that plants show in relation to heavy metal exposure. Thus, it is very important to evaluate the growth and morphology characteristics when considering the plant species or variety to be used for phytoremediation.

The morphological response of the herein tested grain amaranth varieties was characterised by a high level of plant tolerance under elevated Cd stress, demonstrated by the ability to grow and produce new leaves/biomass. However, a reduction in fresh and plant dry weight was observed for “Pribina” and Plainsman (Supplementary Table S1). Out of the three tested varieties, “Zobor” appears to be the most tolerant to Cd contamination, with no significant changes in the production of root and shoot biomass under increasing concentrations of Cd. Although the highest Cd concentration of 15 mg/L did not cause toxic effects, the plants showed a variety-dependent capacity for Cd tolerance. Similar cultivar-dependent Cd tolerance and accumulation has been reported in *A. gangeticus* [31] and in *A. mangostanus* [26].

Phytomanagement can cover the mechanisms of phytoextraction and phytostabilisation. The translocation factor (TF) defines the relationship between the content of the metal in the leaves and its content in the root, providing the information about the efficiency of plant to transport metal(s) from roots to leaves [32]. Therefore, TF determination is important for assessing the plant’s phytoremediation potential. Generally, TF values < 1 indicate that the plant accumulates metal(s) in the roots and rhizomes more than in above-ground biomass [33].

Although the tested amaranths were capable of taking up a significant amount of Cd, the majority of Cd detected by ICP-OES analysis was stored in the root tissues, with only minor distribution into above-ground biomass (Figure 1), with determined TF values < 1 (Figure 2). It has been shown that plant species with small shoot-metal content and with TF < 1 show phytostabilisation potential, and plants with TF > 1, are classified as accumulators/hyperaccumulators [34]. However, most of the plants belong to the group of excluders being able to tolerate heavy metals in the soil with limited translocation of metal(s) to aerial plants [35].

Hypertolerant plants or heavy metal excluders have been studied and plant species with phytostabilisation potential have been identified [34]. Significant uptake and accumulation of Cd in the root tissues with root to shoot translocation factor < 1 have been identified in the aquatic species *Eichhornia crassipes* and *Pistia stratiotes* and grass species *Vetiveria zizanioides* [36,37]. Examples of Cd-excluders also include species like *Oenothera biennis* and *Commelina communis* [38] or *Thlaspi arvense* [39].

As already mentioned, the significant portion of Cd taken up by plants accumulated in the roots (Figure 1). With increasing concentrations of the metal in the hydroponic solution, its concentration in root tissue continuously increased. Unlike roots, the Cd concentration in shoot tissues remained steady regardless of the Cd level in the nutrient solution. Although the tested amaranth varieties cannot be classified as hyperaccumulators because of the inefficient translocation of Cd from roots to shoots (TF <1 for all tested varieties), they can be considered powerful extractors of Cd contamination because of the high biomass production. Nevertheless, high biomass can provide compensation for lower translocation efficiency. For instance, Li et al. [40] tested the phytoextraction capacity of *Amaranthus hypochondriacus* together with *Cichorium intybus*, *Rumex patientia × R. ti-anschanicus*, *Medicago sativa*, *Sorghum bicolor*, and *Sorghum sudanese*. Amaranth showed the greatest amount of extracted Cd among the tested high-biomass forage species. Although the shoot Cd concentrations in amaranth did not meet the criteria for hyperaccumulation, its high biomass compensated for the moderate heavy metal concentrations. Plants having both phytostabilisation and metal-tolerance capacity could potentially be useful for phytoremediation purposes [41]. Thus, amaranth may represent an effective phytostabilisator in the phytomanagement of Cd-loaded soils. Accumulation of high concentrations of Cd in the root system can immobilise the toxic element and protect the surrounding environment from contamination. Cultivation of amaranth on contaminated soils can also positively affect soil features such as pH, soil degradation, and erosion. Similar observations were made by Thongchai et al. [42] using various marigold cultivars, representing a useful tool for the phytomanagement of areas contaminated by Cd.

Another interesting perspective for the implementation of this research is the ability of amaranth to tolerate water-deficient growth conditions [43]. Therefore, amaranth can be used in parallel as vegetation cover and as a heavy metal extractor in semiarid and arid areas where other plant species are not able to grow and reproduce.

Heavy metal exposure can be manifested on the molecular level by alterations in the expression of a set of genes, commonly termed stress genes or stress-associated genes. Over the past decade, genes involved in the metal stress response have been characterised in a number of plant species [44,45,46]. However, there is little knowledge about the effect of metal stress on the expression of genes in the amaranth genome. Therefore, an investigation was performed to determine whether or not Cd exposure can cause alterations in the molecular response of amaranth plants. Five genes previously reported to be associated with (a)biotic stress were selected: pathogenesis-related chitinase 5 (*Chit 5*), *Amaranthus hypochondriacus* important regulator of (a)biotic stress responses in grain amaranth (*Ah24*) [47], the *Amaranthus hypochondriacus* abiotic stress-induced protein DUF642 (*AhDGR2*) [48], phytochelatin synthase which is involved in plant detoxification under Cd stress (*PCS*) [49] and glutamate cysteine ligase as a key component of metal scavenging (*GSH1*) [50].

It is well-known that plant chitinases are involved in responses against phytopathogens [51]. These pathogenesis-related proteins are known to be involved in plant responses to various abiotic stress stimuli such as salinity, wounding, cold, and osmotic stress [52]. They have also been reported to be part of the metal stress defence [53,54,55,56,57].

In response to increasing concentrations of Cd in the nutrient solution, *Chit*5 demonstrated the most remarkable response among the investigated genes in the tested amaranth varieties (Figure 3). Cd stress significantly increased *Chit 5* transcript levels in the amaranth roots. The Plainsman variety showed the highest increase of *Chit 5* gene expression also in shoots pointing to its sensitivity to Cd. Strongly activated *Chit 5* mRNA toward Cd exposure is in accordance with the results reported by Metwally et al. [58] showing relation between metal sensitivity and chitinase expression via higher chitinase transcript levels in more Cd-sensitive *Pisum sativum* genotypes.

A number of crops are significantly responsive to Cd stress via changes in chitinase gene expression and /or chitinase activity, e.g., *Pisum sativum Vicia faba*, *Pisum sativum*, *Hordeum vulgare*, *Zea mays*, *Glycine max*, *Avicennia marina* and *Aegiceras corniculatum* [52,54,55,56,57,58,59]. Although the activities of individual chitinase genes are cultivar- and metal compound-dependent, they are apparently involved in the plant response against heavy metals and metal detoxification in plants [53].

The genes *Ah24* and *AhDGR2* were selected based on previously published results on *Amaranthus hypochondriacus*. Significant induction of the *AhDGR2* gene has been observed under drought and salinity stress [48], and the *Ah24* gene is thought to participate in the defence mechanism against wounding or defoliation [47,60]. Additionally, a proteomic study showed Ah24 accumulation in *A. cruentus* roots in response to salt stress [61]. These findings contribute to the indication that *Ah24* might be an important regulator gene in (a)biotic stress responses in *Amaranthus* spp. [62]. In this study, the expression of *Ah24* in the control and Cd-treated groups was comparable in the “Pribina” and “Zobor” varieties, while the Plainsman variety responded with significantly higher upregulation in the roots of Cd-treated plants compared to leaves (Figure 3).

This pattern of *Chit*5 and *Ah24* gene responses in Plainsman plants indicates its Cd sensitivity. The *AhDGR2* gene was downregulated in both root and shoot tissues of Cd-treated plants across the investigated amaranths. Based on these observations, the involvement of the *AhDGR2* gene in heavy metal stress is ambiguous. It might be speculated as to whether another heavy metal or a higher Cd level would provoke a different response regarding the *AhDGR2* amaranth gene.

One of the three main strategies plants have evolved to minimise the effects of Cd^2+^ stress is the chelation of intracellular Cd^2+^ via cysteine-rich peptides, such as glutathione (GSH) and phytochelatins (PCs) [49]. Glutamate cysteine ligase (GCL) is a key enzyme involved in glutathione biosynthesis and is encoded by the *GSH1* gene [63]. GCL is an essential component of the plant defence system in the detoxification of reactive oxygen species [64,65]. For instance, *Arabidopsis* plants with an identified mutation in the gene encoding the GCL enzyme showed higher susceptibility to biotic stress [66]. Moreover, overexpression of GCL in *Arabidopsis* has been shown to improve tolerance to heavy metals such as Cd and As [67]. In addition, GSH is the precursor for PCs involved in the transport of the PC-Cd complexes into the vacuole [68].

*GSH1* did not show notable alterations under Cd exposure in amaranth plants. However, the *PCS* expression level was upregulated in “Pribina” and in Plainsman roots in which Cd was predominantly accumulated (Figure 3). Ding et al. [69] identified *BnPCS1* as one of four key factors in the response to Cd stress in *Brassica napus* L., as the gene was upregulated after Cd exposure. Recently, remarkably induced *PCS1* gene expression was shown under heavy metal conditions (Cu, Cd and Pb), suggesting a positive relationship between *PCS1* gene expression and the metal-chelating ability of *Solanum lycopersicum* Cd-stressed plants [70]. Thus, *PCS* seemed to be involved in the Cd detoxification pathway in amaranth plants.

## 4. Materials and Methods

### 4.1. Plant Material and Hydroponic Experiments Set Up

Three grain amaranth (*Amaranthus* spp.) varieties have been tested under Cd exposure. Slovak varieties “Pribina” (*A. cruentus*) and “Zobor” (*A. hypochondriacus* x *A. hybridus*) were both previously bred at the home institute by mutation breeding [71,72], and the commercially most preferred variety was Plainsman (*A. hypochondriacus* x *A. hybridus*). Plant growth on soil and hydroponic experiments were performed under controlled conditions in a plant growth chamber (23 °C, 16/8 light/dark cycle, 50% humidity, KK 1450 TOP+FIT model, POL-EKO Aparatura, Poland). First, an initial experiment was performed to assess the optimal Cd concentrations for the study. The following concentrations of Cd were tested: 0, 5, 10, 15, 20, 30 and 40 mg/L according the Zhang et al. [27]. The highest Cd concentrations (30 and 40 mg/L) had a lethal effect on the selected amaranth varieties. A Cd concentration of 20 mg/L was considered as the threshold concentration at which amaranth plants were able to survive; however, detrimental effects of Cd were apparent. For hydroponic experiments, amaranth plants were precultivated in soil until the stage of 4–5 leaves, and then plants at that same stage of development were transferred into the Hoagland hydroponic nutrition solution [73] for 7 days of acclimation. Afterwards, Cd treatment in the form of cadmium chloride was added. For each amaranth variety, independent experiments were established with three Cd concentrations (0 mg/L control), i.e., 5, 10 and 15 mg/L in 5 biological replicates per group. Plants were subjected to Cd treatment for 14 days (in total 21 days in hydroponics: 7 days acclimation + 14 days Cd treatment).

### 4.2. Morphological Measurements

The following plant morphological parameters were determined: (1) root and shoot fresh weight (g) before (day 0) and after (day 21) hydroponics, (2) biomass increment during hydroponics (total fresh plant weight (g) after hydroponics—total fresh plant weight (g) before hydroponics), (3) root and shoot dry mass (g), and (4) root: shoot ratio. Based on the data, overall plant fitness was estimated for control and Cd treated plants. After hydroponics, roots and shoots were separated, dried for 48 h at 50 °C, and then ground into a fine powder and subjected to the ICP-OES analysis.

### 4.3. ICP-OES Analysis

For elemental analysis, the dried plant tissue samples were kept at room temperature until analysis. Each sample (up to 500 mg) was mineralised in a high-performance microwave digestion system (Ethos UP, Milestone Srl, Sorisole, BG, Italy) in a solution of 5 mL HNO_3_ (TraceSELECT^®^, Honeywell Fluka, Morris Plains, USA) and 1 mL H_2_O_2_ (30%, for trace analysis, Merck Suprapur^®^). Samples and a blank sample were digested according to the preloaded method “dried plant tissue” developed by the manufacturer to assure the best results. The method consisted of 15 min of heating at 200 °C, a hold at this temperature for 15 min, followed by 30 min of active cooling. The digests cooled to 50 °C were filtered through Sartorius filter discs (grade 390) (Sartorius AG, Goettingen, Germany) into a volumetric flask and filled up with ultrapure water to a volume of 50 mL.

Analysis of the Cd concentration was carried out using an inductively coupled plasma optical emission spectrophotometer (ICP Thermo ICAP 7000 Dual, Thermo Fisher Scientific, Waltham, Massachusetts, USA). The operating conditions were: plasma RF power 1150 W, purge gas flow 3.20 L/min, auxiliary gas flow 0.50 L/min, coolant gas flow 12 L/min, nebuliser gas flow 0.40 L/min, nebuliser gas pressure 120 kPa, pump speed 50 rpm. The detection limit of Cd for a dilution factor of 1 was 0.00042 μg/L. Multielement standard solution V for ICP (Sigma-Aldrich Production GmbH, Switzerland) was used for calibration and the accuracy of Cd determination was assessed using certified reference material (ERM-CD281, Sigma-Aldrich Production GmbH, Switzerland).

### 4.4. Gene Expression Analysis

#### 4.4.1. RNA Isolation and Reverse Transcription

Root and leaf tissues were collected from the control and Cd (15 mg/L) treated amaranth plants and immediately flash frozen in liquid nitrogen for further analysis. Total RNA was isolated using the TRI reagent procedure [74]. Briefly, 50 mg of plant tissue was ground using the TissueLyzer and 0.5 mL of TRIZOL reagent was added to each sample. Samples were incubated at room temperature for 10 min, centrifuged at 12,000 rpm for 15 min and supernatant was transferred in to the new 1.5 mL tube. All centrifugation steps were carried out at 4 °C in the prechilled centrifuge. Then, 0.1 mL of chloroform was added; samples were vortexed and centrifuged at 12,000 rpm for 15 min. Supernatant was transferred into the new tube, and RNA precipitated using 0.25 mL of isopropyl alcohol, then samples were left at room temperature for 30 min and centrifuged at 12,000 rpm for 10 min. The supernatant was removed and an RNA wash was performed using 75% ethanol. The RNA pellet was air dried and resuspended in 50 µL of DEPC-treated water. The quality and quantity of RNA was checked using a Nanodrop spectrophotometer and the RNA integrity was verified on a 1.5% agarose gel. The cDNA was synthesised from 1 µg of RNA using a Maxima First Strand cDNA Synthesis Kit for RT-qPCR (Thermo Fisher Scientific; Waltham, Massachusetts, USA) according the manufacturer’s instructions.

#### 4.4.2. qRT-PCR Assay of Stress Responsive Genes

Changes in the expression of five stress responsive genes in experiments involving Cd exposure were quantified using quantitative real-time PCR analysis: chitinase 5 (*Chit 5*; Phytozome AHYPO_015445-RA), gene encoding a DUF642 protein (*AhDGR2*; Phytozome AHYPO_ 022469-RA), *Ah24* (NCBI GenBank JN384107.1), phytochelatin synthase (*PCS1*; Phytozome AHYPO_009753-RA), and glutamate cysteine ligase (*GSH1*; Phytozome AHYPO_009268-RA).

Primer sequences for *AhDGR2* and *Ah24* were selected according to previously published work [47,48]. To design primers for chitinase 5, phytochelatin synthase and glutamate cysteine ligase, known sequences from the NCBI database were blasted against the sequenced genome of *A. hypochondriacus* in Phytozome, and then conserved gene regions were used for primer design. The following primers were used for selected genes: *Chit 5* For 5′-CGGTACCGTTTCAGTCCCAA-3′, Rev 5′-AGAGGATCCACCCGTACCAA-3′; *AhDGR2* For 5′-CCCTTCCTGGTTGGATGGTC-3′, Rev 5′-CAACTTGAGCAATGGCGCTT-3′; *Ah24* For 5′-GATTTAATTGAGATGGCTGAAA-3′, Rev 5′-GTTAGTGCAGCTTGTTCGC-3′; *PCS* For 5′-TGCGCAAGAATTGACAAGCC-3′, Rev 5′-AGGGTCCAGAAATTGGTCGT-3′; *GSH1* For 5′-CCGGTGTGAAGCTGTATGGA-3′, Rev 5′-CAGCAACCACTGGATCGTCT-3′. The *Amaranthus hypochondriacus Actin* (*AhACT*) gene was used as an internal standard in all experiments. For *AhACT*, the primers according to Palmeros-Suárez et al. [48] were used: for 5′-CGTGACCTGACTGATTACCTTA-3′ and Rev 5′-GCTCGTAGTTCTTCTCAATGGC-3′.

Firstly, standard curves were generated using a series of five-fold cDNA dilutions (1:1, 1:5, 1:25, 1:125, 1:625 and 1:3125). For each gene, the PCR efficiency (E) was determined, and the correlation coefficient (*R*^2^) was calculated using linear regression. A PCR efficiency of 90%–110% and *R*^2^ > 0.99 were accepted. Quantitative PCR (qPCR) was performed using a LightCycler^®^ Nano (Roche). The reaction mixtures consisted of 2x SsoAdvanced Universal SYBR^®^ Green supermix (Bio-Rad), 400 nM of each forward and reverse primer, 50 ng of cDNA, and nuclease-free water added up to the total reaction volume of 10 µL. A two-step amplification protocol was applied, with initial denaturation at 95 °C for 30 s, 45 cycles of denaturation at 95 °C for 15 s and annealing/polymerisation at 60 °C for 60 s; a melt analysis was then performed from 60 °C to 97 °C at 0.1 °C/s to verify the specificity of the desired amplicon. The expression of all analysed genes was determined in each reaction using the threshold cycle (Ct value). The Ct value was set automatically by the LightCycler Nano software. The calculation of relative gene expression was performed according Pfaffl [75] using the PCR efficiencies and the Ct values of control and unknown samples.

### 4.5. Data Analysis and Statistics

Statistical analysis of the obtained data was carried out using Statistica 10 software (StatSoft Inc., Tulsa, OK, USA). One-way ANOVA followed by the LSD post hoc test was performed in order to find statistical differences among the means. Paired sample t-test was applied to compare expression of analysed genes between roots and shoots of tested varieties. The relationship between the Cd concentration and TF was assessed by Pearson’s correlation coefficient.

## 5. Conclusions

Three grain amaranth varieties, including newly bred “Pribina” and “Zobor”, were tested for their response towards Cd exposure and their ability to accumulate Cd in order to be potentially used in phytoremediation. Our results showed that the tested varieties could tolerate used Cd concentrations without any lethal effects; however, plant dry weight was reduced at the highest Cd level. The varieties were capable of absorbing a high level of Cd, predominantly in the roots, with limited root-to-shoot translocation. Therefore, they are appropriate for effective phytostabilisation of Cd-polluted soils. Nevertheless, most suitable for possible phytomanagement of Cd polluted soils was found variety “Pribina”, showing significantly highest Cd uptake with increasing Cd level. In addition, the gene expression patterns of chitinase (*Chit5*) and phytochelatin synthase (*PCS1*) in roots and shoots demonstrated that the variety “Pribina” was the most Cd-tolerant among the three tested varieties. 

Our results indicate a clear role of chitinase (*Chit5*), a typical stress-related defence component, in the amaranth response to Cd exposure. The involvement of *PCS1* in the alleviation of Cd stress might be required in Cd-tolerant amaranth plants.

Further studies assessing, e.g., the intracellular distribution of Cd and performing a detailed characterisation of genes and transcript levels under Cd stress might shed more light onto the physiological and molecular mechanism of Cd accumulation and tolerance in grain amaranth.

## Supplementary Materials

The following are available online at https://www.mdpi.com/2223-7747/9/5/572/s1, Supplementary Table S1: Plant growth measurements under Cd stress and ICP-OES determination of Cd in root/shoot tissues of investigated amaranth varieties.
